# Effects of Two Commercial Electronic Prescribing Systems on Prescribing Error Rates in Hospital In-Patients: A Before and After Study

**DOI:** 10.1371/journal.pmed.1001164

**Published:** 2012-01-31

**Authors:** Johanna I. Westbrook, Margaret Reckmann, Ling Li, William B. Runciman, Rosemary Burke, Connie Lo, Melissa T. Baysari, Jeffrey Braithwaite, Richard O. Day

**Affiliations:** 1 Centre for Health Systems and Safety Research, Australian Institute of Health Innovation, Faculty of Medicine, University of New South Wales, Sydney, Australia; 2 School of Psychology, Social Work & Social Policy, University of South Australia, Adelaide, Australia; 3 Pharmacy Department, Concord Repatriation General Hospital, Sydney, Australia; 4 Australian Institute of Health Innovation, Faculty of Medicine, University of New South Wales, Sydney, Australia; 5 Centre for Clinical Governance Research, Australian Institute of Health Innovation, Faculty of Medicine, University of New South Wales, Sydney, Australia; 6 Department of Clinical Pharmacology and Toxicology, St Vincent's Hospital, Sydney, and Faculty of Medicine, University of New South Wales, Sydney, Australia; Edinburgh University, United Kingdom

## Abstract

**Background:**

Considerable investments are being made in commercial electronic prescribing systems (e-prescribing) in many countries. Few studies have measured or evaluated their effectiveness at reducing prescribing error rates, and interactions between system design and errors are not well understood, despite increasing concerns regarding new errors associated with system use. This study evaluated the effectiveness of two commercial e-prescribing systems in reducing prescribing error rates and their propensities for introducing new types of error.

**Methods and Results:**

We conducted a before and after study involving medication chart audit of 3,291 admissions (1,923 at baseline and 1,368 post e-prescribing system) at two Australian teaching hospitals. In Hospital A, the Cerner Millennium e-prescribing system was implemented on one ward, and three wards, which did not receive the e-prescribing system, acted as controls. In Hospital B, the iSoft MedChart system was implemented on two wards and we compared before and after error rates. Procedural (e.g., unclear and incomplete prescribing orders) and clinical (e.g., wrong dose, wrong drug) errors were identified. Prescribing error rates per admission and per 100 patient days; rates of serious errors (5-point severity scale, those ≥3 were categorised as serious) by hospital and study period; and rates and categories of postintervention “system-related” errors (where system functionality or design contributed to the error) were calculated. Use of an e-prescribing system was associated with a statistically significant reduction in error rates in all three intervention wards (respectively reductions of 66.1% [95% CI 53.9%–78.3%]; 57.5% [33.8%–81.2%]; and 60.5% [48.5%–72.4%]). The use of the system resulted in a decline in errors at Hospital A from 6.25 per admission (95% CI 5.23–7.28) to 2.12 (95% CI 1.71–2.54; *p*<0.0001) and at Hospital B from 3.62 (95% CI 3.30–3.93) to 1.46 (95% CI 1.20–1.73; *p*<0.0001). This decrease was driven by a large reduction in unclear, illegal, and incomplete orders. The Hospital A control wards experienced no significant change (respectively −12.8% [95% CI −41.1% to 15.5%]; −11.3% [−40.1% to 17.5%]; −20.1% [−52.2% to 12.4%]). There was limited change in clinical error rates, but serious errors decreased by 44% (0.25 per admission to 0.14; *p* = 0.0002) across the intervention wards compared to the control wards (17% reduction; 0.30–0.25; *p* = 0.40). Both hospitals experienced system-related errors (0.73 and 0.51 per admission), which accounted for 35% of postsystem errors in the intervention wards; each system was associated with different types of system-related errors.

**Conclusions:**

Implementation of these commercial e-prescribing systems resulted in statistically significant reductions in prescribing error rates. Reductions in clinical errors were limited in the absence of substantial decision support, but a statistically significant decline in serious errors was observed. System-related errors require close attention as they are frequent, but are potentially remediable by system redesign and user training. Limitations included a lack of control wards at Hospital B and an inability to randomize wards to the intervention.

*Please see later in the article for the Editors' Summary*

## Abstract 2

In a before-and-after study, Johanna Westbrook and colleagues evaluate the change in prescribing error rates after the introduction of two commercial electronic prescribing systems in two Australian hospitals.

## Editors' Summary

**Background:**

Medication errors—for example, prescribing the wrong drug or giving a drug by the wrong route—frequently occur in health care settings and are responsible for thousands of deaths every year. Until recently, medicines were prescribed and dispensed using systems based on hand-written scripts. In hospitals, for example, physicians wrote orders for medications directly onto a medication chart, which was then used by the nursing staff to give drugs to their patients. However, drugs are now increasingly being prescribed using electronic prescribing (e-prescribing) systems. With these systems, prescribers use a computer and order medications for their patients with the help of a drug information database and menu items, free text boxes, and prewritten orders for specific conditions (so-called passive decision support). The system reviews the patient's medication and known allergy list and alerts the physician to any potential problems, including drug interactions (active decision support). Then after the physician has responded to these alerts, the order is transmitted electronically to the pharmacy and/or the nursing staff who administer the prescription.

**Why Was This Study Done?:**

By avoiding the need for physicians to write out prescriptions and by providing active and passive decision support, e-prescribing has the potential to reduce medication errors. But, even though many countries are investing in expensive commercial e-prescribing systems, few studies have evaluated the effects of these systems on prescribing error rates. Moreover, little is known about the interactions between system design and errors despite fears that e-prescribing might introduce new errors. In this study, the researchers analyze prescribing error rates in hospital in-patients before and after the implementation of two commercial e-prescribing systems.

**What Did the Researchers Do and Find?:**

The researchers examined medication charts for procedural errors (unclear, incomplete, or illegal orders) and for clinical errors (for example, wrong drug or dose) at two Australian hospitals before and after the introduction of commercial e-prescribing systems. At Hospital A, the Cerner Millennium e-prescribing system was introduced on one ward; three other wards acted as controls. At Hospital B, the researchers compared the error rates on two wards before and after the introduction of the iSoft MedChart e-prescribing system. The introduction of an e-prescribing system was associated with a substantial reduction in error rates in the three intervention wards; error rates on the control wards did not change significantly during the study. At Hospital A, medication errors declined from 6.25 to 2.12 per admission after the introduction of e-prescribing whereas at Hospital B, they declined from 3.62 to 1.46 per admission. This reduction in error rates was mainly driven by a reduction in procedural error rates and there was only a limited change in overall clinical error rates. Notably, however, the rate of serious errors decreased across the intervention wards from 0.25 to 0.14 per admission (a 44% reduction), whereas the serious error rate only decreased by 17% in the control wards during the study. Finally, system-related errors (for example, selection of an inappropriate drug located on a drop-down menu next to a likely drug selection) accounted for 35% of errors in the intervention wards after the implementation of e-prescribing.

**What Do These Findings Mean?:**

These findings show that the implementation of these two e-prescribing systems markedly reduced hospital in-patient prescribing error rates, mainly by reducing the number of incomplete, illegal, or unclear medication orders. The limited decision support built into both the e-prescribing systems used here may explain the limited reduction in clinical error rates but, importantly, both e-prescribing systems reduced serious medication errors. Finally, the high rate of system-related errors recorded in this study is worrying but is potentially remediable by system redesign and user training. Because this was a “real-world” study, it was not possible to choose the intervention wards randomly. Moreover, there was no control ward at Hospital B, and the wards included in the study had very different specialties. These and other aspects of the study design may limit the generalizability of these findings, which need to be confirmed and extended in additional studies. Even so, these findings provide persuasive evidence of the current and potential ability of commercial e-prescribing systems to reduce prescribing errors in hospital in-patients provided these systems are continually monitored and refined to improve their performance.

**Additional Information:**

Please access these Web sites via the online version of this summary at http://dx.doi.org/10.1371/journal.pmed.1001164.

- ClinfoWiki has pages on medication errors and on electronic prescribing (note: the Clinical Informatics Wiki is a free online resource that anyone can add to or edit)
- Electronic prescribing in hospitals challenges and lessons learned describes the implementation of e-prescribing in UK hospitals; more information about e-prescribing in the UK is available on the NHS Connecting for Health Website
- The Clinicians Guide to e-Prescribing provides up-to-date information about e-prescribing in the USA
- Information about e-prescribing in Australia is also available
- Information about electronic health records in Australia

## Introduction

It is well over a decade since electronic prescribing systems were first shown to reduce medication errors [1],[2], demonstrating their potential to address this long-standing, costly problem [3]–[5]. However, recent reviews [6]–[9] reveal that many questions remain unanswered regarding the extent to which systems deliver improvements in medication safety in different settings, important contextual and work practice factors associated with effectiveness, and the cost benefit of systems. To date, evidence of effectiveness rests largely on the experiences of a few hospitals using home-grown systems.

A central question is whether commercial e-prescribing systems can deliver the same benefits as home-grown systems. There is little work comparing commercial systems or the interactions between system design and error rates and types, despite increasing concerns regarding new errors associated with their use [8],[10],[11]. Implementation of these organisation-wide clinical information systems is complex [12],[13] with a multitude of work process and cultural factors [14]–[16], which affect system adoption and use, driving both intended and unintended outcomes [10],[11],[17],[18].

In 2011, the US Agency for Healthcare Research and Quality [8] released a review of the effects of health information technology on medication management and drew attention to the need for research that evaluates systems in everyday settings and allows comparisons between systems and study sites. Our aim was to evaluate two commercial e-prescribing systems with respect to their effectiveness in reducing prescribing errors and their propensities for introducing new types of error.

## Methods

### Sample and Data Collection

**Figure 1:**
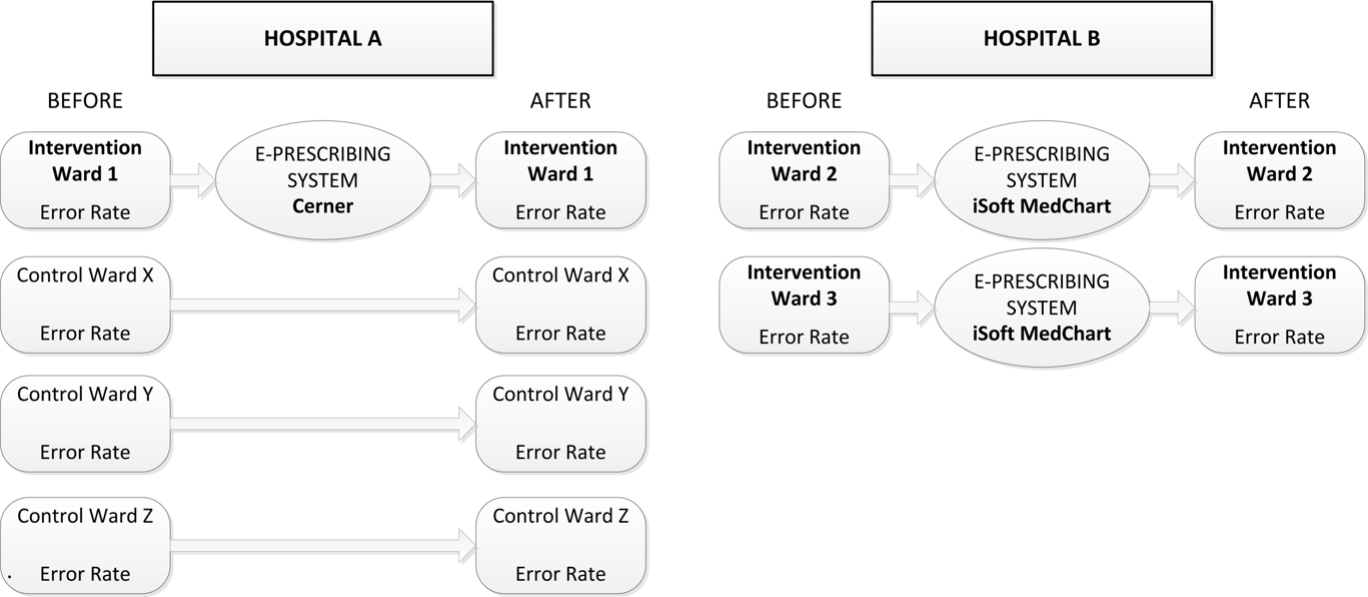
Outline of study design.

A before and after study design was implemented at two major teaching hospitals in Sydney, Australia. Hospital A had 400 beds and Hospital B 326 beds. At Hospital A data were collected from four wards pre and post e-prescribing system implementation (two geriatric, a renal/vascular, and a respiratory ward). One ward (geriatric) was assigned the intervention and the remaining three wards acted as controls. At Hospital B the intervention was implemented on two wards (psychiatry and cardiology), and error rates were evaluated in the pre and post e-prescribing implementation periods. Figure 1 outlines the study design.

A daily review of all inpatient medication charts (*n* = 3,291) was conducted by three pharmacists independent from the hospitals for at least two months pre- and postintervention, with the exception of the psychiatric ward (1 mo pre and post). Data collection at Hospital A was conducted between May–August 2006 (pre) and May–August 2008 (28 wk post e-prescribing system), and at Hospital B between November 2007–March 2008 (pre), and March 2008–February 2010 (16 and 10 wk post system introduction). Data collection was dictated by the hospitals' e-prescribing system implementations, which experienced several delays. Human research ethics approval was received from both hospitals and the University of Sydney.

### Error Classification

Errors were classified into procedural (three categories) or clinical errors (14 categories) (Table S1 lists error definitions). Prescribing errors identified in the intervention wards in the postperiod were additionally reviewed to assess whether or not they were “system-related” (see definitions Table S1). System-related errors were defined as errors where system functionality or design contributed to the error, and there was little possibility that another cause, such as a lack of knowledge, produced the error. For example, an order for an inappropriate drug located on a drop-down menu next to a likely drug selection was flagged as a system-related error. Thus all system-related errors underwent dual classification in terms of (1) their manifestation according to one of the 17 procedural or clinical error categories and (2) the system-related mechanism that was deemed to be associated with those errors. In this paper, the system-related errors are reported according to their clinical manifestation and are listed in a separate table, as strategies for their prevention are likely to relate to system redesign or improved functionality.

Inter-rater reliability tests were conducted at regular intervals and compared pharmacist reviewers' agreement with respect to number and type of errors. These tests involved double audit of 10% of all admissions and produced kappa scores of 0.82–0.84. In the last stage of the research, 1,097 admissions (33% of the total sample) were re-reviewed in order to ensure consistency of data collection between the early and later data collection periods. Two pharmacists independently rated the actual or potential severity of errors (Box 1); disagreement was settled by consensus with input from a clinical pharmacologist (ROD) when required. Severity review committees involving an emergency physician, hospital pharmacists, and nurses from both hospitals were also given subsets of errors to classify during the study.

**Box 1. Severity Assessment Code [47]**

***Minor errors***

1. Insignificant: Incident is likely to have little or no effect on the patient.

2. Minor: Incident is likely to lead to an increase in level of care e.g. review, investigations, or referral to another clinician.

***Serious errors***

3. Moderate: Incident is likely to lead to permanent reduction in bodily functioning, increased length of stay, surgical intervention.

4. Major: Incident is likely to lead to a major permanent loss of function.

5. Serious: Incident is likely to lead to death.

## Hospital Prescribing and the Interventions

In the preintervention period all wards used paper medication charts in which the prescribing doctors wrote orders. These charts were then used by nursing staff as the medication administration charts. There was no intermediate transcription step between a prescriber's order and the final medication chart entry, as is the case in some countries.

Ward pharmacy services were provided during the weekdays but not on weekends. The research pharmacists' daily review of the medication charts may have occurred either before or after the ward pharmacists had done their rounds. All interventions (corrections) made by the ward pharmacists in patients' medication charts were identifiable and noted (i.e., errors detected by the ward pharmacists were included in the study).

Interventions consisted of the implementation of two e-prescribing systems (Cerner Millennium PowerOrders and iSoft MedChart) integrated with each hospitals' computerised order entry system. Prescribers were required to use the systems to prescribe medications in the post period.

Hospital A implemented the Cerner system, where prescribing is mainly by menu selection of pre-prepared order sentences that are triggered upon drug selection and that can be modified by the prescriber. “Care sets” allow for a group of related orders to be selected and ordered simultaneously with a single click. Unlisted medications and prescribing order comments need to be generated by the prescriber. In the Cerner e-prescribing system, active decision support at the time of study consisted of allergy alerts and drug–drug interaction alerts set at the most severe level (using the Multum database). Medication orders could not be completed if the patient's allergy status was not recorded. If a prescriber wished to over-ride an alert they needed to select an override reason from a drop-down menu or enter a free-text comment. Passive decision support included a drug information database, the highly structured order sentences, and predefined order-sets such as the palliative care set. Further passive decision support allowed prescribers a diabetic medication view, an anticoagulant view, and an analgesic view, which provided integration of patients' lab results and drug doses.

Hospital B implemented the iSoft MedChart system. Prescribing could be completed in three ways following selection of a drug: (1) long-hand, where prescribing information is entered via drop-down lists or free text boxes; (2) “quicklists,” or prewritten orders; and (3) “protocols,” where common combinations of prewritten orders can be selected.

MedChart included alerts for allergy checking, pregnancy warnings, therapeutic duplication, some dose-range checking, and a number of local decision-support rules (such as drug and therapeutics committee decisions and antibiotic stewardship guidelines). Drug–drug interaction alerts were not operational during the study. All alerts allowed the prescriber to continue with the order. Alerts were all “pop-ups” on the screen. Approximately half of the alerts were for information only; prescribers were not required to take action and just had to close the alert box. Others required the prescriber to respond by ticking an “override” box. For approximately 10% of the alerts prescribers were required to enter a free-text reason for overriding the alert in order to proceed. Drug information references were available online as passive decision support.

During the intervention periods both sites used paper orders for a small subset of medications. At Hospital A, heparin infusions and patient-controlled analgesia remained on paper charts.

At Hospital B, orders for intravenous (IV) fluids, IV infusions (e.g., heparin infusion), variable dose regimes (such as titrated or reducing doses), insulins, oral anticoagulants (warfarin), chemotherapy, parenteral nutrition, and epidural or patient-controlled analgesia remained on paper charts. The prescriber was required to order an electronic prompt to signal the administration times for these drugs, but the actual drug orders were located on a paper chart. Errors related to these electronic prompts were included in the postperiod data collection.

## Statistical Analysis

The error data were linked with the patient admission data, which matched the study periods. Rates of prescribing errors per admission and per 100 patient days were calculated for each error type and category, by period (pre/post), group (intervention/control), hospital, and ward. Serious errors (graded≥3) (Box 1) were examined by group, error type, and period. System-related error rates per admission were examined for both systems. The 95% CIs for the average error rates per admission and per 100 patient days were calculated using the large sample approximation of mean ±1.96× standard error. For the pre- and postanalysis, two-sample *t*-tests were used to compare baseline data with post e-prescribing system data with the level of significance set at 5%. The 95% CIs for percentage changes were calculated as per the Fieller CI [19]. All statistical analyses were carried out with SAS 9.2 [20].

## Results

### Incidence, Type, and Severity of Prescribing Errors at Baseline

The 1,923 admissions across the six wards reviewed at baseline revealed 11,168 prescribing errors, an average of 5.8 per admission. The majority (*n* = 8,225; 73.6%; 4.28 per admission) were procedural (e.g., unclear, incomplete, or illegible orders) with the remaining 26.4% (*n* = 2,943; 1.53 per admission) comprising clinical errors. Hospital A had higher procedural and clinical error rates at baseline compared to Hospital B (Table 1). The rates of serious errors were comparable (respectively, 0.28 per admission; 95% CI 0.22–0.35; *n* = 296 versus 0.26 per admission; 95% CI 0.21–0.31; *n* = 226).

**Table 1:** Summary of baseline prescribing error rates by hospital.

Error Category	Hospital A, 1,045 Admissions	Hospital B, 878 Admissions
Procedural error rate	5.63 (5.01–6.26); *n* = 5,888	2.66 (2.43–2.90); *n* = 2,337
Clinical error rate	2.01 (1.73–2.30); *n* = 2,104	0.96 (0.84–1.07); *n* = 839
Total error rate	7.65 (6.83–8.47); *n* = 7,992	3.62 (3.30–3.93); *n* = 3,176

Errors/admission (95% CI); *n*, number of errors.

Error rates for individual wards within hospitals were similar at baseline (Tables 2 and 3). The four most frequent clinical error types in each ward were also considerably similar. At baseline, duplicate therapy and wrong dose/volume errors appeared in the top four most frequent errors for all wards. “Legal/procedural” was the most frequent procedural error category on all wards.

**Table 2:** Prescribing error rates per admission by hospital, ward type, error category, and error type at baseline.

Error	Error Category	Hospital A								Hospital B			
		Intervention Ward 1		Control Ward X		Control Ward Y		Control Ward Z		Intervention Ward 2		Intervention Ward 3	
		*n*	Per Adm	*n*	Per Adm	*n*	Per Adm	*n*	Per Adm	*n*	Per Adm	*n*	Per Adm
**Procedural errors**	Legal/procedural	392	2.24	647	2.70	566	2.80	1,312	3.07	123	1.60	1,220	1.52
	Incomplete order	379	2.17	548	2.28	462	2.29	1,024	2.39	112	1.45	588	0.73
	Unclear order	85	0.49	115	0.48	82	0.41	276	0.64	26	0.34	268	0.33
	**Total**	856	4.89	1310	5.46	1,110	5.50	2,612	6.10	261	3.39	2,076	2.59
**Clinical errors**	Duplicated therapy	56	0.32^a^	86	0.36^a^	76	0.38^a^	156	0.36^a^	20	0.26^a^	156	0.19^a^
	Wrong strength	48	0.27^a^	42	0.18	42	0.21^a^	120	0.28^a^	1	0.01	51	0.06
	Wrong dose/volume	35	0.20^a^	96	0.40^a^	64	0.32^a^	216	0.50^a^	21	0.27^a^	121	0.15^a^
	Wrong rate/frequency	23	0.13^a^	71	0.30^a^	23	0.11	92	0.21	3	0.04^a^	55	0.07
	Wrong route	20	0.11	34	0.14	40	0.20^a^	100	0.23	28	0.36^a^	64	0.08
	Wrong drug	14	0.08	23	0.10	15	0.07	76	0.18	0	0	43	0.05
	Drug not prescribed	9	0.05	45	0.19^a^	37	0.18	144	0.34^a^	1	0.01	137	0.17^a^
	Drug–drug interaction	8	0.05	12	0.05	6	0.03	36	0.08	1	0.01	83	0.10^a^
	Not indicated	7	0.04	4	0.02	11	0.05	32	0.07	0	0	3	0.00
	Wrong timing	5	0.03	13	0.05	20	0.10	68	0.16	0	0	24	0.03
	Wrong formulation	4	0.02	0	0	13	0.06	4	0.01	0	0	3	0.00
	Inadequate monitoring	4	0.02	4	0.02	3	0.01	16	0.04	1	0.01	8	0.01
	Allergy	4	0.02	4	0.02	6	0.03	16	0.04	0	0	15	0.02
	Wrong patient	1	0.01	0	0	0	0	0	0	0	0	0	0
	**Total**	238	1.36	434	1.81	356	1.76	1,076	2.51	76	0.99	763	0.95

Adm, the number of admissions; *n*, number of errors.

a Indicates the four most frequent error types in each ward.

### Changes in Prescribing Error Rates Following E-prescribing System Implementation

Total error rates fell significantly (*p*<0.0001) in each intervention ward following e-prescribing system implementation: by 66.1% (95% CI 53.9%–78.3%) in intervention ward 1; 57.5% (33.8%–81.2%) intervention ward 2; and 60.5% (48.5%–72.4%) intervention ward 3. The three Hospital A control wards experienced small decreases in prescribing error rates per admission, none of which were statistically significant, (respectively −12.8% [95% CI −41.1% to 15.5%] control ward X; −11.3% [−40.1% to 17.5%] control ward Y; and −20.1% [−52.2% to 12.4%] control ward Z). Table 3 reports error rates in the pre- and postperiods for all wards.

A marked reduction in procedural errors drove this decline. In the intervention ward at Hospital A the procedural error rate fell by 90.2% (from 4.89 per admission to 0.48), and at Hospital B by 93.6% (from 2.66 per admission to 0.17). Hospital A had significantly higher procedural error rates at baseline and a difference between the sites persisted in the postperiod. The rates of clinical prescribing errors did not significantly change with the exception of intervention ward 2 where there was a significant increase in clinical error rate: from 0.99 to 1.70 per admission (*p* = 0.04) (Table 3).

**Table 3:** Comparison of prescribing error rates pre- and postelectronic prescribing system implementation.

Ward	Period	Adm	Prescribing Error Rates per Admission									Prescribing Errors per 100 Patient Days
			Procedural Errors			Clinical Errors			Total Errors			Total Errors Mean (95% CI)
			*n*	Mean (95% CI)	*p*	*n*	Mean (95% CI)	*p*	*n*	Mean (95% CI)	*p*	
**Hospital A**												
**Intervention 1**	**Pre**	175	856	4.89 (4.02–5.76)	<0.0001	238	1.36 (1.08–1.64)	0.2	1,094	6.25 (5.23–7.28)	<0.0001	51.6 (43.0–60.3)
	**Post**	164	78	0.48 (0.37–0.58)		270	1.65 (1.28–2.01)		348	2.12 (1.71–2.54)		17.3 (13.0–21.6)
**Control X**	**Pre**	240	1,310	5.45 (4.58–6.34)	0.3	434	1.81 (1.49–2.13)	0.2	1,744	7.27 (6.23–8.31)	0.2	78.1 (63.5–92.7)
	**Post**	236	1,141	4.83 (3.91–5.76)		356	1.51 (1.17–1.85)		1,497	6.34 (5.20–7.49)		65.7 (55.6–75.9)
**Control Y**	**Pre**	202	1,110	5.49 (4.57–6.42)	0.2	356	1.76 (1.41–2.11)	0.9	1,466	7.25 (6.12–8.39)	0.3	60.3 (48.7–71.8)
	**Post**	135	629	4.66 (3.83–5.48)		241	1.79 (1.41–2.16)		870	6.44 (5.39–7.50)		64.8 (46.4–83.3)
**Control Z**	**Pre**	428	2,612	6.10 (4.77–7.44)	0.3	1,076	2.51 (1.88–3.15)	0.06	3,688	8.62 (6.82–10.42)	0.1	123.1 (92.3–154.0)
	**Post**	368	1,884	5.12 (4.09–6.15)		625	1.77 (1.34–2.21)		2,536	6.89 (5.55–8.23)		101.3 (72.4–130.2)
**Hospital B**												
**Intervention 2**	**Pre**	77	261	3.39 (2.47–4.31)	<0.0001	76	0.99 (0.59–1.38)	0.04	337	4.38 (3.30–5.45)	<0.0001	39.4 (31.3–47.4)
	**Post**	64	10	0.16 (0.06–0.25)		109	1.70 (1.13–2.27)		119	1.86 (1.27–2.45)		10.2 (6.2–14.2)
**Intervention 3**	**Pre**	801	2,076	2.59 (2.35–2.83)	<0.0001	763	0.95 (0.83–1.08)	0.07	2,839	3.54 (3.21–3.88)	<0.0001	48.7 (39.9–57.5)
	**Post**	401	69	0.17 (0.12–0.23)		493	1.23 (0.96–1.50)		562	1.40 (1.11–1.69)		17.5 (13.9–21.0)

Includes system-related errors (*n* = 358), which occurred in the intervention wards in the postperiod.

Adm, number of admissions; *n*, number of errors.

Prescribing error rates per 100 patient days confirmed a significant decline in total error rates. As Table 3 shows, intervention ward 1 experienced a 66.5% decline in error rates from 51.6 to 17.3 per 100 patient days; intervention ward 2, a 74.1% reduction, and intervention ward 3, a 64.1% reduction.

### Changes in the Rates of Serious Prescribing Errors Following E-prescribing System Implementation

We examined the number of serious errors (i.e., severity≥3) per admission in the intervention wards and Hospital A control wards in each period. There was a significant 44% serious error rate reduction (*p* = 0.0002) in the intervention wards following system implementation (Table 4). The Hospital A control wards experienced no significant change (16.7% reduction; *p* = 0.4).

**Table 4:** Serious errors per admission by study group and period.

Error Type	Period	Control				Intervention			
		Adm	*n*	Error per Adm (95% CI)	*p*	Adm	*n*	Error per Adm (95% CI)	*p*
**Procedural**	Pre	870	25	0.03 (0.01–0.05)	0.4	1,053	81	0.08 (0.05–0.10)	<0.0001
	Post	739	30	0.04 (0.02–0.06)		629	3	0 (0–0.01)	
**Clinical**	Pre	870	234	0.27 (0.20–0.34)	0.3	1,053	182	0.17 (0.14–0.21)	0.1
	Post	739	157	0.21 (0.15–0.28)		629	84	0.13 (0.10–0.17)	
**Total**	Pre	870	259	0.30 (0.22–0.37)	0.4	1,053	263	0.25 (0.21–0.29)	0.0002
	Post	739	187	0.25 (0.18–0.32)		629	87	0.14 (0.10–0.18)	

Adm, number of admissions; *n*, number of serious errors.

### Changes in Categories of Prescribing Errors Post E-prescribing System Implementation Excluding System-Related Errors

We examined changes in the categories of errors in the intervention wards and Hospital A control wards with system-related errors removed (Table 5), and then examined the ways in which system-related errors manifested themselves at each hospital (Table 6). In the postperiod there were substantial changes in the procedural error rates in the intervention wards, with unclear, incomplete, and legal/procedural orders almost eliminated (90.8% reduction for Hospital A and 93.6% for Hospital B, *p*<0.0001), while there was little change in these categories in the Hospital A control wards (Table 5).

**Table 5:** Prescribing errors by type, category, hospital, and period for the intervention and control wards.

Error Category	Control Wards Combined				Hospital A				Hospital B			
	Pre		Post		Pre		Post		Pre		Post	
	*n*	Error Rate per Adm	*n*	Error Rate per Adm	*n*	Error Rate per Adm	*n*	Error Rate per Adm	*n*	Error Rate per Adm	*n*	Error Rate per Adm
Legal/procedural	2525	2.90	1726	2.34	392	2.24	69	0.42	1343	1.53	76	0.16
Incomplete order	2034	2.34	1622	2.19	379	2.17	3	0.02	700	0.80	2	0.00
Unclear order	473	0.54	306	0.41	85	0.49	2	0.01	294	0.33	0	0.00
**Total**	**5032**	**5.78**	**3654**	**4.94**	**856**	**4.89**	**74**	**0.45**	**2337**	**2.66**	**78**	**0.17**
**Clinical errors**												
Duplicated therapy	318	0.37	173	0.23	56	0.32	52	0.32	176	0.20	28	0.06
Wrong strength	204	0.23	102	0.14	48	0.27	1	0.01	52	0.06	4	0.01
Wrong dose/volume	376	0.43	187	0.25	35	0.20	24	0.15	142	0.16	69	0.15
Wrong rate/frequency	186	0.21	110	0.15	23	0.13	16	0.10	58	0.07	29	0.06
Wrong route	174	0.20	147	0.20	20	0.11	1	0.01	92	0.10	29	0.06
Wrong drug	114	0.13	56	0.08	14	0.08	3	0.02	43	0.05	4	0.01
Drug not prescribed	226	0.26	128	0.17	9	0.05	18	0.11	138	0.16	35	0.08
Drug–drug interaction	54	0.06	88	0.12	8	0.05	11	0.07	84	0.10	29	0.06
Not indicated	47	0.05	6	0.01	7	0.04	8	0.05	3	0.00	3	0.01
Wrong timing	101	0.12	192	0.26	5	0.03	5	0.03	24	0.03	120	0.26
Wrong formulation	17	0.02	9	0.01	4	0.02	4	0.02	3	0.00	1	0.00
Inadequate monitoring	23	0.03	7	0.01	4	0.02	4	0.02	9	0.01	10	0.02
Allergy	26	0.03	42	0.06	4	0.02	8	0.05	15	0.02	3	0.01
Wrong patient	0	—	2	0.00	1	0.01	0	—	0	0.00	0	0.00
**Total**	**1,866**	**2.14**	**1249**	**1.69**	**238**	**1.36**	**155**	**0.95**	**839**	**0.96**	**364**	**0.78**

Excludes 358 system-related prescribing errors that occurred in the intervention wards in the post period (See Table 6 for further details of these).

**Table 6:** The manifestation of system-related prescribing error rates by type and hospital.

Error Category	Hospital A			Hospital B		
	*n*	Percent of Errors	Rate per Adm	*n*	Percent of Errors	Rate per Adm
Wrong route	27	23	0.16	1	0	0
Wrong drug	14	12	0.09	10	4	0.02
Prompt not ordered	13	11	0.08	28	12	0.06
Wrong formulation	11	9	0.07	31	13	0.07
Not indicated	11	9	0.07	5	2	0.01
Wrong dose	11	9	0.07	0	0	0
Wrong ancillary info	11	9	0.07	17	7	0.04
Wrong rate/frequency	8	7	0.05	25	10	0.05
Wrong strength	5	4	0.03	106	44	0.23
Wrong dose unit	4	3	0.02	14	6	0.03
Incomplete order	4	3	0.02	1	0	0
Duplicated order	0	0	0	1	0	0
**Total**	119	100	0.73	239	100	0.51

The intervention wards also experienced greater changes in the rates of specific categories of prescribing errors compared to the Hospital A control wards. In the control wards (at Hospital A) the most notable changes were a doubling in the rates of wrong timing errors (from 0.12 to 0.26 per admission) and drug–drug interaction errors (0.06 to 0.12). However, there were also considerable reductions in the rates of duplicate therapy errors (0.37 to 0.23) and wrong dose/volume errors (0.43 to 0.25 per admission) (Table 5).

We examined changes in rates of error category by hospital to assess any potential impact of specific system functionality (Table 5). Hospital B experienced a considerably larger increase in the rate of timing errors (0.03 errors/admission to 0.26) than the intervention ward (0.3 pre and post) or control wards (0.12 to 0.26) at Hospital A.

There was some evidence of the effect of the limited decision support in the e-prescribing system at Hospital B, with a marked decline in duplicate therapy error rates (0.20–0.06 per admission; 70% reduction) compared to both the Hospital A control wards (0.37–0.23; 38% reduction) and the intervention ward at Hospital A (0.32 pre and post; no change). Allergy alerts were enabled at both sites but there was little change in allergy error rates, which remained low in both periods (Table 5).

High level drug–drug interaction alerts were enabled at Hospital A but there was no evidence of a significant decrease in these errors (0.05–0.07). Hospital A had marked reductions in wrong strength errors (0.27–0.01; 96% reduction) and wrong route errors (0.11–0.01; 91%) in the intervention ward. Hospital B, in addition to the decline in duplicate therapy errors, experienced the largest declines in rates of wrong strength (0.06–0.01; 83%) and “drug not prescribed” errors (0.16–0.08; 50%) (Table 5).

### System-Related Prescribing Errors by Hospital

Each of the hospitals experienced prescribing errors associated with the use of the new systems. Combined, the intervention wards experienced 0.57 system-related errors per admission, which accounted for 34.8% (358/1,029) of all prescribing errors in these wards in the postperiod.

Nearly all system-related prescribing errors manifested as clinical errors (99%, *n* = 353). The clinical error rate (including system-related errors) for the intervention wards increased from 1.02 (*n* = 1,077) to 1.39 (*n* = 872) per admission following e-prescribing system implementation. If system-related clinical errors were removed this rate fell to 0.83 (*n* = 519) in the postperiod, representing a significant reduction (*p* = 0.03) in clinical error rate. Thus, system-related errors were a major reason for the e-prescribing system not delivering a significant reduction in the overall rate of clinical errors (Table 3).

The rate and categories of system-related errors differed by hospital. At Hospital A these errors occurred at a rate of 0.73 (95% CI 0.53–0.92) per admission and on the two wards at Hospital B 0.75 (95% CI 0.44–1.06) and 0.48 (95% CI 0.36–0.60). A low percentage of these system-related errors were serious errors (3%; *n* = 11).

Table 6 shows the distribution of “system-related” errors across error categories by hospital. Hospital A had higher rates of seven error types compared to Hospital B. System-related errors that resulted in wrong strength errors were markedly higher at Hospital B (0.23 per admission versus 0.03 at Hospital A).

## Discussion

Both commercial e-prescribing systems were associated with a statistically significant reduction in total prescribing error rates by over 55%, driven by the substantial reductions in incomplete, illegal, and unclear orders. While there was little change in the rate of clinical errors for the intervention wards (and an increase in one intervention ward), the rate of serious prescribing errors decreased by 44% relative to the Hospital A control wards, which experienced a decline of 17%. Thus, while these e-prescribing systems with limited decision support were not associated with a substantial reduction in the rate of clinical errors, they were associated with a reduction in some of the most potentially serious errors.

Other studies have evaluated home-grown e-prescribing systems. For example, Bates et al. [2] reported a 55% reduction in serious nonintercepted medication errors (prescribing, dispensing, and administration errors) following the introduction of a home-grown system, although, as they had no control wards the change attributable to the e-prescribing system could not be determined. Major difficulties in comparing effectiveness studies of e-prescribing systems have been consistently highlighted [8],[9],[21].

Although both systems in our study had only limited decision support enabled, there was some evidence that this was effective in reducing some error types. For example, the MedChart system had duplicate therapy alerts and was associated with a fall in these error rates, consistent with other studies [22]–[28] of decision-support interventions. However, designing effective organisational-wide decision support is challenging [29]–[36]. Additional research at one of the study sites has, for example, shown that during ward rounds the effectiveness of the decision support is compromised, as the senior clinicians making the prescribing decisions were seen to instruct junior clinicians on the round to enter the orders. Alerts received were thus not seen by the decision-makers and the doctors entering the orders ignored most alerts received during this process [37]. Responses to decision support alerts outside ward rounds, particularly at night by junior doctors, may be quite different. There remains much to understand about how decision support can be integrated into clinical work processes and lead to safer and more effective prescribing.

An important starting point is to obtain baseline data of the incidence and severity of prescribing errors to facilitate the design of targeted decision support. Few organisations have such data and rarely are prescribers provided with feedback regarding errors. Behaviour change is unlikely in such situations. e-prescribing systems provide enormous capacity to provide real-time feedback of prescribing behaviours; this should be examined together with efforts to embed decision support and alerts.

The increases in wrong timing errors found in the control wards in Hospital A are likely to be attributable to a new paper-based standard national inpatient medication chart, which was introduced in the postperiod. This new chart required specific timing information from prescribers and compliance was modest, an effect noted at other Australian hospitals [38]. Timing errors also increased substantially in the intervention wards at Hospital B. These errors are likely to be associated with the design of the e-prescribing system, which required prescribers to modify the default administration times when necessary. For example, with an order for metformin (500 mg tablet, dose 500 mg oral in the morning), the timing defaults to 0800, and the local rule in the e-prescribing system states that prescribers should change this default time to 0700 (breakfast time at the hospital) because the drug is an oral hypoglycaemic and should be taken with food. Timing errors were logged when prescribers failed to change such default times. This situation was in contrast to the e-prescribing system at Hospital A where administration times were linked to specific order sentences. For example, the order sentence for the metformin example above would be: metformin 500 mg, oral, tab, mane (morning) after food. The “mane after food” defaults the time to 0730 (breakfast time at the hospital), thus avoiding a potential timing error.

There was a high rate of system-related errors for both hospitals accounting for 35% of prescribing errors in the intervention wards in the postperiod. Without these system-related errors, the overall clinical error rate in the intervention wards would have declined significantly in the postperiod. The types of system-related errors varied considerably by hospital, likely due to differences in system designs and the structuring of prescribing tasks. Work is underway to examine the relationships between specific system functionalities and types of system-related errors. For example, the disparity in the rates of system-related errors resulting in “wrong strength” errors at Hospital B (0.23 per admission) compared to Hospital A (0.03), and the rate of “wrong route” errors at Hospital A (0.16 per admission) compared to almost none at Hospital B, suggest specific system features that predispose to these error types. Such findings provide a focus for examining the redesign of system features and/or training of prescribers, and more generally the degree to which such systems reflect ways of working within these clinical environments.

While several studies [10],[11],[39] have described types of system-related errors, few have systematically classified them and quantified their occurrence or severity. Their high volume indicates that they should be targeted; our experience suggests that a high proportion is amenable to remediation through minor system redesign, such as listing the most frequently used option first on drop-down menus, or creating prestructured orders to reduce the need for users to construct complex order sentences. Where system changes cannot be made, areas for targeted training can be identified [40]. This illustrates the importance of identifying what errors are occurring, and when, and highlights the improvements that can be achieved once these types of errors are reduced. Hospitals must allocate sufficient resources to detect and respond to such issues as they arise [41].

Beyond answering the central question regarding the effectiveness of e-prescribing systems in reducing errors, the study has produced comprehensive data on prescribing errors in hospitals in the absence of these systems, with longitudinal data across three control wards in Hospital A. The findings showed considerable similarities in error rates at baseline despite the very different clinical areas represented, from geriatrics to cardiac surgery and psychiatry. This suggests that the underlying mechanisms of prescribing errors are generic rather than speciality specific. There was no substantial change in error rates in the control wards over an average of 2 y, notwithstanding the fact that medication errors were targeted by a range of interventions during this time, including the introduction of a standard national medication inpatient chart designed to reduce errors [38]. These findings confirm how difficult it is to reduce medication error rates and are consistent with the findings of the EPOC Cochrane collaboration series, which demonstrate the relative ineffectiveness of conventional initiatives in changing clinical practice [42]. It also highlights the value of e-prescribing systems in achieving the outcomes they did.

The complexity of undertaking “real-world” studies should not be underestimated [43]–[45]. The research was subject to substantial delays in system implementation at both sites. The postimplementation data collection periods were different at the two sites and it is possible that this time difference influenced the results. We consulted with clinical and other staff at the sites to seek advice about the required “settling in” period prior to postintervention data collection. At Hospital B, which had the shorter postintervention periods, the system had already been implemented on several other wards and thus many problems had been dealt with in these earlier implementations. There is limited evidence from other studies to clearly identify the effects of time from intervention to outcome measurements and this should be a consideration for future studies.

We were unable to randomise our intervention wards, and because of a change in implementation plans we were unable to obtain a control ward at Hospital B. The availability of three control wards at Hospital A proved to be a major strength given potential confounders such as other safety initiatives that may have impacted prescribing error rates. We had no control over the selection of the intervention wards. At Hospital A, intervention ward 1 was the first ward in the hospital to use the system and one factor in ward selection was a willing clinician leader. At Hospital B several wards had the e-prescribing system implemented before the study intervention wards. The study had a wide range of specialties represented and this was a potential additional challenge for comparison, but the baseline prescribing error rates by type across the wards suggest that specialty was not strongly associated with any particular error type. Some wards, such as the psychiatry ward, would have had a narrower range of drugs prescribed than on other wards. We are confident of the quality of our data due to the extensive inter-rater reliability testing applied throughout the study.

This study provides persuasive evidence of the current and potential value of commercial e-prescribing systems to significantly and substantially reduce prescribing errors in hospital in-patients. However, as other studies have demonstrated [40],[43],[44], success in achieving this outcome is dependent upon many contextual and organisational factors and multimethod studies are of great value in order to understand the mechanisms by which e-prescribing systems impact upon prescribing behaviours [12]. Our qualitative studies at the study sites revealed clinicians' greatest concern regarding the introduction of e-prescribing systems was the associated work practice changes [46], and qualitative and observational studies may best identify the nature of these changes. Experience has shown that embedding systems into everyday practice is a long-term project [13]. Importantly, the results highlight the need to continually monitor and refine the design of these systems to increase their capacity to improve both the safety and appropriateness of medication use in hospitals.

## Supporting Information

Alternative Language Abstract S1 Chinese translation of the abstract by LL.
(DOCX)

Table S1 Definitions of prescribing error categories used in the study.
(DOCX)
