# Supplementary material for: Effects of Two Commercial Electronic Prescribing Systems on Prescribing Error Rates in Hospital In-Patients: A Before and After Study
Source: PLoS Med. 2012 Jan 31;9(1):e1001164. doi: 10.1371/journal.pmed.1001164 (PMC3269428; doi:10.1371/journal.pmed.1001164)
Supplement: Alternative Language Abstract S1 — Chinese translation of the abstract by LL. (DOCX) [file pmed.1001164.s001.docx]

**两个电子商业处方系统在减少处方错误率方面的有效性: 一项系统实施之前和之后的比较研究**

**摘要
背景**：许多国家大量地投资商业电子处方系统, 但是目前很少有人研究并评估它在减少处方错误率方面的有效性。尽管使用电子处方系统引发的新错误越来越多被关注, 我们还是缺乏对系统设计和错误之间的相互关系的了解。 这项研究评估了两个商业电子处方系统在减少处方错误率方面的有效性，及产生新错误类型的倾向。

**方法**：我们做了一项电子处方系统实施之前和实施之后的比较研究，涉及两个澳大利亚的教学医院, 3291住院医疗记录, 其中1923从使用电子处方系统之前和1368从使用之后。医院A的一个病房使用了Cerner Millennium系统, 其它三个病房没有实施电子处方系统, 也就是控制病房。在医院B, 两间病房实施了iSoft MedChart系统。 我们进行了之前和之后的错误率比较。我们鉴别了开处方程序（例如不清楚，不完整的处方）和临床（如错误剂量，错药）的错误。按医院和研究期间(之前和之后), 我们计算了每例入院和每百天患者住院处方错误率, 严重的错误率, 以及系统使用相关的错误率和类型。

**结果**：三个使用了电子处方系统的病房的错误率在统计意义上显著减少（分别减少66.1％（95％CI: 53.9％ - 78.3％）; 57.5％（33.8％-81.2％） ;和60.5％（48.5％-72.4％））。医院A每例入院的错误率从6.25个（95％CI: 5.23 - 7.28）下降至2.12（95％CI: 1.71 - 2.54，P <0.0001），医院B从3.62（95％CI: 3.30 - 3.93）降为1.46（95％CI: 1.20 - 1.73，P <0.0001）。不清楚的、不合理的和不完整的处方大量减少是错误率下降的主要原因。而没有使用系统的病房的处方错误率没有显著变化（分别为-12.8％（95％CI为-41.1％-15.5％）; -11.3％（-40.1％-17.5％），-20.1％（-52.2％-12.4％））。临床处方错误率有限，但严重的临床错误减少了44％（每例入院0.25到0.14，P = 0.0002）, 没有使用系统的病房减少了17％ (0.30至0.25，P = 0.40）。这两个医院都有与使用系统相关的错误（每例入院0.73个和0.51个），占系统使用后总错误的35％; 两个系统产生的与使用系统相关的错误类型各不相同。

**结论**：这些商业电子处方系统的实施显著减少处方错误率。在缺少决策支持的情况下, 临床错误率的减少是有限的，但严重的错误在统计意义上显著地下降。与实施系统相关的错误则需要密切关注，尽管它们频繁发生，但系统重新设计和用户培训可以避免这类错误。这项研究的缺陷包括医院B没有控制病房和无法随机选择干预病房。
