# Supplementary material for: Effects of Two Commercial Electronic Prescribing Systems on Prescribing Error Rates in Hospital In-Patients: A Before and After Study
Source: PLoS Med. 2012 Jan 31;9(1):e1001164. doi: 10.1371/journal.pmed.1001164 (PMC3269428; doi:10.1371/journal.pmed.1001164)
Supplement: Table S1 — Definitions of prescribing error categories used in the study. (DOCX) [file pmed.1001164.s002.docx]

Table S1 Definitions of prescribing error categories used in the study

| **CLINICAL ERRORS** | | |
| --- | --- | --- |
| **Error category** | **Definition** | **Examples** |
| Wrong drug | Occurs when an inappropriate medication or IV fluid is prescribed  e.g. the drug prescribed is not indicated for the patient’s condition; the drug or IV fluid is contraindicated for a coexisting condition; or an IV drug is prescribed with an incompatible diluent  Note: Excludes generic substitution | e.g. **hydrocortisone 25mg oral mane** was prescribed instead of cortisone;  **chamomile lotion** was ordered instead of calamine lotion |
| Wrong dose/volume | Occurs when the prescribed medication dose or IV fluid volume is higher or lower than that recommended for the condition, taking into account the patient’s age, weight, renal and liver function  May also occur when a dose is not altered in response to abnormal drug serum levels or laboratory tests  Note: A dose may differ from normal recommended reference ranges and not be classed as an error where it is accepted practice to do so, i.e. the dose may have been queried by a pharmacist, but the specialist physician insisted on the prescribed dose, e.g. high dose flucloxacillin despite severe renal impairment in patients with severe infection when recommended by the infectious diseases team; low doses of tricyclic antidepressants initiated by the pain team. |  |
| Wrong rate/frequency | Occurs when the prescribed frequency of administration of a drug or an IV rate falls outside the recommended range |  |
| Wrong route | Occurs when a medication is prescribed via an incorrect route of administration | e.g. IV medication was prescribed orally;  left eye was written instead of right eye |
| Wrong formulation | Occurs when the wrong dosage form of a medication is ordered | e.g. an immediate release tablet was prescribed when an extended release form was required |
| Wrong timing | Occurs when a drug is prescribed at the wrong time of day | e.g. simvastatin prescribed in the morning instead of the evening (it is more efficacious when taken at night) |
| Wrong strength | Occurs when the prescribed drug strength is incorrect; the concentration of an IV infusion is prescribed incorrectly; or a dose is prescribed that does not exist or would not be able to be obtained easily from the current dose forms | e.g. mg was prescribed instead of micrograms (or vice versa)  e.g. **alendronate 75mg tab oral, take one tab once weekly** (weekly dose only available as 70mg tablets) |
| Wrong patient | Occurs when a medication is prescribed for the wrong patient   e.g. the prescriber writes a drug order intended for patient A on the medication chart belonging to patient B |  |
| Not prescribed | Occurs when a medication clinically indicated for the patient is not prescribed; or the drug is not reordered when the patient’s medications are recharted |  |
| Not indicated | Occurs when a drug which is not indicated is prescribed for the patient; a drug is continued following a clinically significant adverse drug reaction; a drug which is no longer indicated is reordered; or a drug which should have been discontinued has not been ceased  May also occur when a prescriber fails to cease/withhold a drug in response to abnormal drug serum levels or laboratory tests | e.g. fluticasone/ salmeterol inhaler prescribed for a patient without chronic obstructive airways disease  e.g. an antibiotic which was not discontinued after completion of the course |
| Duplicated drug therapy | Occurs when two orders have been prescribed for one medication and both orders are active; there are two active orders for the same medication on two different charts; or the same drug is prescribed twice, as a single agent and as a combination product  May also occur when two drugs are prescribed for the same indication when only one is necessary | e.g. one order was prescribed by generic and one by brand name  e.g. ranitidine and omeprazole for gastro-oesophageal reflux disease |
| Drug-drug interaction | Occurs if two of the drugs prescribed for a patient are known to have a clinically significant interaction and this interaction is not acknowledged and monitored |  |
| Allergy | Occurs when a drug is prescribed for a patient with a documented clinically significant allergy to that drug/class of drugs |  |
| Inadequate monitoring | Occurs when the prescriber fails to order appropriate and timely clinical or laboratory tests to assess the patient’s response to prescribed therapy  Note: if adequate lab tests are ordered, but the results are not acted upon accordingly, resulting in potential or actual compromised patient care, this may be classed as wrong dose/volume error |  |
| **PROCEDURAL ERRORS** | | |
| Unclear order | Occurs when the prescription is unclear or ambiguous  e.g. the writing is illegible; or the order contains additional comments which apparently contradict the medication order | e.g. **clotrimoxazole topical interdigital BD** (the prescriber was confused between cotrimoxazole and clotrimazole) |
| Incomplete order | Occurs when the order does not include all the necessary information i.e. drug name; strength (if appropriate); formulation (if appropriate); dose; route of administration; frequency ; the diluent for injectables; duration of time and/or rate of infusion (IV infusions); duration of time (IV fluids) |  |
| Legal/Procedural | Occurs when an aspect related to the prescription does not comply with the law, the NSW Department of Health or the hospital policy (and has not been assigned as an unclear order); the allergy field of the medication chart has not been completed; or the strength, dose, route or frequency of an existing handwritten medication order has been altered (such a change legally requires the entire order to be recharted) |  |
| **Additional System-related Error Categories** | | |
| System-related errors were defined as errors where e-prescribing system functionality or design contributed to the error, and there was little possibility that another cause, such as lack of knowledge, produced the error. For example, an order for an inappropriate drug located on a drop-down menu next to a likely drug selection was flagged as a system-related error. | | |
| Wrong dose unit | Occurs when an inappropriate term is used as a unit of dose  Examples include:  unit, dose, sachet (Cerner) i.u. (international unit), oral liquid, infusion, injection, tablet, mouthwash, cream (MedChart) | e.g. **Movicol 1 oral liquid** (for 1 sachet); **potassium clavulanate/ ticarcillin (0.1g, 3g) 3.1 infusion** (for 3.1g);  **everolimus 0.25mg, 0.25 tablet** (intended dose 1x 0.25mg tablet) (MedChart)  e.g. **potassium chloride (Chlorvescent) 2U** (for 2 tablets);  **loperamide 2 unit** (for 2 capsules) (Cerner) |
| Prompt not ordered | Occurs when a required ancillary prompt is not ordered  Note: Dermal patches e.g. glyceryl trinitrate (GTN), nicotine and fentanyl were mostly ordered in Cerner as a care set (order set) consisting of two separate (unlinked) orders, one for application and the second for removal of the patch. An active order for a dermal patch could be prescribed without a corresponding order for its removal.  Warfarin was prescribed in Cerner as a care set consisting of an order for warfarin and an (unlinked) ancillary prompt for the prescriber to check the INR value against the selected warfarin target range (warfarin check).  In iSoft MedChart, orders for insulins, warfarin, heparin infusions or variable dose regimens such as titrated or ^r^educing doses remained on paper charts, and the prescriber also ordered an electronic prompt appropriately timed to fall due whenever the paper chart order fell due. | e.g. a missing dermal patch removal order (Cerner)  e.g. a missing warfarin check (INR) prompt (Cerner)  e.g. a missing paper chart prompt (MedChart) |
| Wrong ancillary information | Occurs when additional information attached to an order, such as an order comment or ancillary product information, is inconsistent with correct information contained in the order;  May also occur when a prescriber selects an existing order sentence intended for nurse initiated orders (Cerner) | e.g. IV order with instructions to swallow whole;  an oral order with instructions for IV infusion;  **nystatin drops 200 000units (1mL)** (200 000 units is contained in 2mL);  **GTN patch 10mg/24hour, 1 patch mane, order comment: 50mg/24hour** (Cerner)  e.g. **lisinopril 5mg mane, order comment: half a 5mg tab** (dose was increased from 2.5 to 5mg, but the order comment was not changed);  **temazepam 10-20mg PRN, order comment: minimum dosage interval 10 hours** (MedChart) |

Abbreviations: PRN = as required; stat = immediately; BD = twice a day; TDS = three times a day; mane = (in the) morning; midi = at midday; nocte = (at) night; qXh = every X hours; mcg/hr = microgram per hour; i.u. = international unit
